# The efficacy of imagery in the rehabilitation of people with Parkinson’s disease: protocol for a systematic review and meta-analysis

**DOI:** 10.1186/s13643-022-02041-z

**Published:** 2022-08-08

**Authors:** Tabitha Singer, Paul Fahey, Karen P. Y. Liu

**Affiliations:** 1 School of Health Sciences, Western Sydney University, Penrith, NSW Australia; 2 Translational Health Research Institute, Western Sydney University, Penrith, NSW Australia

**Keywords:** Parkinson’s disease, Imagery, Rehabilitation, Activities and participation, Systematic review, Meta-analysis

## Abstract

**Background:**

Parkinson’s disease (PD) is a neurodegenerative disorder of the nervous system that affects movement. Individuals with PD commonly experience difficulty initiating movements, slowness of movements, decreased balance, and decreased standing ability. It has been shown that these motor symptoms adversely affect the independence of individuals with PD. Imagery is the cognitive process whereby a motor action is internally reproduced and repeated without overt physical movement. Recent studies support the use of imagery in improving rehabilitation outcomes in the PD population. However, these data have inconsistencies and have not yet been synthesised. The study will review the evidence on the use of imagery in individuals with PD and to determine its efficacy in improving rehabilitation outcomes.

**Methods:**

Randomised controlled clinical trials comparing the effects of imagery and control on activities, body structure and function, and participation outcomes for people with PD will be included. A detailed computer-aided search of the literature will be performed from inception to June 2021 in the following databases: MEDLINE, EMBASE, CINAHL, PsycINFO, Cochrane Library, Web of Science, and Scopus. Two independent reviewers will screen articles for relevance and methodological validity. The Physiotherapy Evidence Database (PEDro) scale will be utilised to evaluate the risk of bias of selected studies. Data from included studies will be extracted by two independent reviewers through a customised, pre-set data extraction sheet. Studies using imagery with comparable outcome measures will be pooled for meta-analysis using the random effect model with 95% CI. If individual studies are heterogeneous, a descriptive review will analyse variance in interventions and outcomes. A narrative data analysis will be considered where there is insufficient data to perform a meta-analysis.

**Discussion:**

Several studies investigating imagery in the PD population have drawn dissimilar conclusions regarding its effectiveness in rehabilitation outcomes and clinical applicability. Therefore, this systematic review will gather and critically appraise all relevant data, to generate a conclusion and recommendations to guide both clinical practice and future research on using imagery in the rehabilitation of people with PD.

**Funding:**

This research received no specific grant from any funding agency in the public, commercial, or not-for-profit sectors.

**Systematic review registration:**

PROSPERO registration number CRD42021230556.

## Background

### Description of condition

Parkinson’s disease (PD) is a chronic, progressive, neuro-degenerative disease, characterised by a break-down of the cells of the substantia nigra—the area of the brain largely responsible for planning and controlling body movements [1, 2]. All individuals with PD experience different symptoms at different times with varying severity. Early-stage PD is characterised by unilateral and mild symptoms such as a slight tremor [3]. Due to its progressive nature, mid-stage PD typically presents with a significant slowing of body movements and slightly decreased balance, posture, and gait. Advanced-stage PD is distinguished by severe symptoms, limited or no walking and standing ability, rigidity, and bradykinesia (slowness of movements, difficulty initiating movements) [4].

These motor symptoms affect the three levels of functioning according to the International Classification of Functioning (ICF): body structure and function, activities, and participation [5–7]. They impact independence and an individual’s ability to perform their meaningful everyday activities [6, 8].

### Description of intervention

Imagery may be a suitable non-pharmacologic treatment for such decline in individuals with PD. It can be classified into two categories, visual and kinesthetic imagery [9]. Both involve imagination or visualisation. In visual imagery, an individual will visualise moving a limb without the actual sensing of the muscles. During kinesthetic imagery, the person imagines the muscle movement for an action. In a therapeutic context, imagery is the cognitive process whereby a particular motor action or kinesthetic experience is internally reproduced (visualised or ‘imagined’) and repeated extensively without any physical movement [10]. Imagery can be used to promote one’s learning or enhancing of a motor skill or general wellbeing. During imagery, actions can be viewed internally (the individual views the task execution from a first-person perspective) or externally (the individual ‘views’ themselves executing task from a third-person perspective) [11]. Imagery can also be externally cued-guided by a therapist or other, or internally cued, by the individual themselves. For example, imagery may involve an individual dividing motor actions into single steps and imagining these single steps from their own perspective or through ‘viewing’ themselves completing the actions.

### How the intervention might work

The theory behind imagery is that imagining an action shares the same neural mechanisms as unconscious motor preparation [10]. It has also been proven that imagined and executed actions share the same neural structures and recruit overlapping brain regions [12]. Thus, repeated practicing of imagined motor tasks is as effective as physical practice of motor tasks.

The way this happens is not fully understood. But it is hypothesised that both psychological and physiological mechanisms are involved [13]. The psychological mechanism is suggested to be improving the cognitive elements of skills, including breaking an action down into steps, attentional focus, and promoting learning of movement strategies by exploring different execution patterns. The theorised physiological mechanisms include neural changes in the central nervous system, like greater relaxation and altered programming of the motor system itself [14]. Therefore, individuals with PD who perform imagery in a therapeutic context may experience improvements in motor planning and motor action execution.

### Importance of doing this review

Imagery has been proven effective in other neurological conditions including stroke [15–19]. There are a handful of studies showing that imagery can have positive effects in rehabilitation in PD [20, 21]. These changes include improved arm-hand ability, performance of activities of daily living, cognition, and motivation. New research has provided initial evidence on the positive use of imagery in PD rehabilitation when used in combination with other therapies, including action observation [22, 23].

Whilst there is a growing body of evidence highlighting the effectiveness of imagery in individuals with PD, there are conflicting data in current literature. Tremblay, Leonard [24] found that study participants with PD could not engage in observation and imagery as effectively as healthy controls. However, other studies indicate individuals with PD can successfully engage in imagery. One such study found no significant difference in completion times of imagined and physical tasks in participants with PD in an ‘ON’ medication state [25].

To date, there is no synthesised evaluation of the literature regarding the efficacy of imagery in PD rehabilitation. Da Silva, de Sales [26] offer comprehensive descriptions of imagery protocols in PD in their systematic review. Further research to determine the efficacy of these protocols is needed to bridge this gap in knowledge.

## Objective

The objective of this study is to gather and synthesise current research on the use of imagery in individuals with PD and to determine its efficacy in improving rehabilitation outcomes as classified by the categories of the ICF [5].

## Methods

The protocol was developed according to the Preferred Reporting Items of Systematic Reviews and Meta-Analyses Protocol (PRISMA-P) guidelines [27] and has been registered on PROSPERO database (Ref: CRD42021230556).

### Criteria for selecting studies for this review

#### Type of studies

Randomised controlled clinical trials comparing one group undergoing treatment with imagery and a control group will be considered eligible for the present study.

#### Types of participants

Studies involving individuals with a diagnosis of PD (any sex, age, stage of disease progression) will be included. Studies that administer imagery to individuals with PD with dementia or other diseases, such as Alzheimer’s disease, stroke, or multiple sclerosis, will be excluded.

#### Types of interventions

Studies addressing the treatment of individuals with PD using imagery protocols with a focus on the outcomes of interest will be selected. Imagery was defined as the cognitive process of internally reproducing or ‘imagining’ a motor action repeatedly without any physical movement. Interventions requiring specialised equipment (including electro-myographic stimulation or virtual reality technologies) and those associated with medication beyond the participants’ habitual medications will not be included in the present systematic review. For the control groups, both active and passive control types will be considered.

#### Types of outcome measures

Studies that report results related to ‘body structure and function’ (i.e. muscle strength and cognition), ‘activities’ (i.e. activities of daily living, gait and mobility), and ‘participation’ (i.e. quality of life) using standardised or non-standardised assessments before and after the intervention or follow-up will be included. The primary outcome measures of interests will be activities of daily living performance under the ‘activities’ category. It includes self-care such as feeding and dressing and instrumental activities of daily living such as meal preparation and doing laundry. The secondary outcome measures include gait and walking in the ‘activities’ category such as functional mobility and measures capturing walking speed or cadence, motor and cognitive function in the ‘body structure and function’ category such as balance, attention and concentration, memory, visuospatial ability and executive function and quality of life measures in the ‘participation’ category. These categories have been selected as they are the three domains of functioning in the ICF [5] and thus have the capacity to represent a holistic perspective on rehabilitation.

### Search strategy for identification of studies

#### Electronic searches

We will conduct electronic searches in the Medical Literature Analysis and Retrieval System Online (MEDLINE) (via OVID), Embase Biomedical Answers (Embase) (via OVID), Web of Science, The Cochrane Library, Cumulative Index to Nursing and Allied Health Literature (CINAHL) (via Ebscohost), Scopus and PsycINFO (via Ebscohost). The search strategy was created considering terms related to the main outcomes of interest. A combination of search terms and Medical Subject Heading terms will be used (‘Parkinson disease’ or ‘parkinsonism’, ‘Parkinson’ or ‘hypokinesia’, ‘imagery’, ‘guided imagery’ or ‘imagination’, ‘motor imagery’, ‘mental imagery’, ‘simulated movement’ or ‘visuomotor imagery’, ‘rehabilitation’ (Table 1 in Appendix for a sample search). A systematic literature search strategy will be conducted from inception to June 2021.

#### Search of other sources

We will perform a hand search of the reference lists of the studies included in the review to identify any potentially relevant studies not retrieved during the electronic search. The grey literature will not be searched.

### Data collection and analysis

#### Selection of studies

**Fig. 1:**
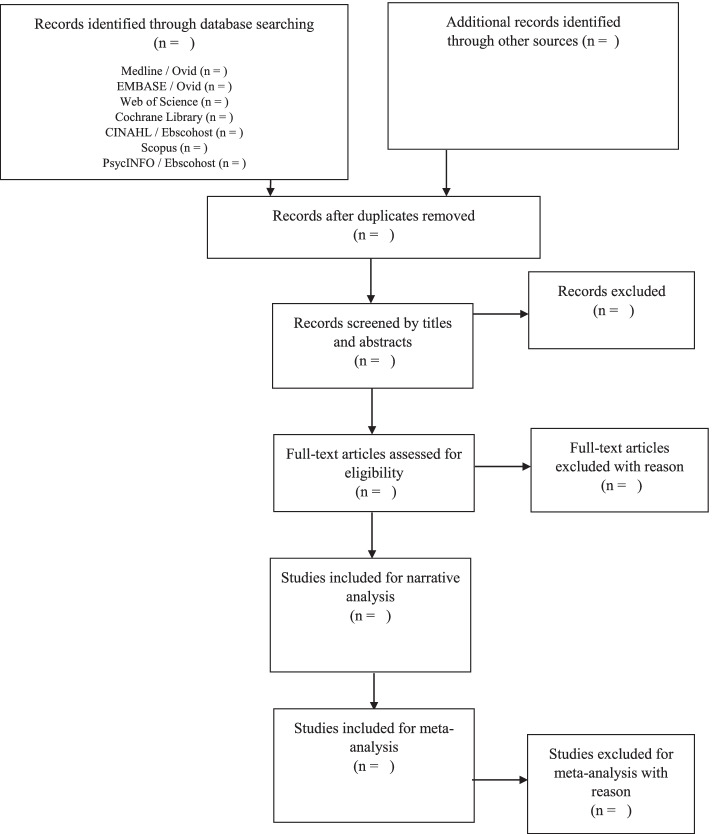
Flow diagram of the study selection process based on the PRISMA guidelines

We follow the PRISMA guidelines in the study selection (Fig. 1). Databases will be searched by one reviewer (TS) to identify potential titles and abstracts. Two independent reviewers (KL and TS) will screen the titles and abstracts of the publications retrieved during the electronic search based on the eligibility criteria. Potentially relevant studies will then undergo full-text analysis. The entire selection process will be performed by consensus. If no consensus can be reached on a given study, a third reviewer will be consulted for the final decision. We will use the Covidence Review during the selection of the studies (www.covidence.org). Covidence is a systematic review software which assists in title and abstract screening, full-text screening and extracting study characteristics.

#### Data extraction

After the selection of the studies, the two reviewers (TS and KL) will work independently. A data extraction form will be developed by the research team and piloted independently by two reviewers on 10% of the identified studies and modified as required prior to use. The following information will be extracted from each study: country/setting, study design, sample size, sex of participants, mean stage of PD, mean disease duration characteristics of participants; intervention and control details, number of sessions and frequency of treatment, outcome measures and major findings. The intended outcomes will include activities of daily living performance, gait and walking, motor and cognitive function and quality of life measures. Disagreements concerning data extraction will be resolved through discussion. A third reviewer will be consulted where a consensus cannot be reached.

#### Assessment of risk of bias

Two independent reviewers (TS and KL) will utilise the Physiotherapy Evidence Database (PEDro) scale to assess the risk of bias [28]. The PEDro is scored out of 10, as item 1 is not included in score calculation as it represents external validity. Any disagreements between reviewers will be resolved through discussion. A third reviewer will be consulted where a consensus cannot be reached. We will acknowledge and report on concerns of bias that can influence the outcome of this review, in particular selection bias, performance bias and publication bias. Studies with a total score less than 50% of the maximum are considered to have low methodological quality [28].

#### Measures of treatment effect

For studies with comparable outcome measures, data will be pooled for meta-analysis. Continuous data will be presented as 95% confidence interval (CI) and mean difference (MD). For dichotomous data, the risk ratio (RR) and 95 % CI will be calculated. Furthermore, the number needed to treat (NNT) will be calculated.

#### Dealing with missing data

In case of missing data, authors will be contacted to provide further information. If authors fail to provide information within two months, intention-to-treat analysis will be used for the extrapolated data. The impact of this will be reported in the discussion section of the systematic review.

#### Assessment of heterogeneity

Heterogeneity will be addressed by pooling studies which investigate the same intervention and outcomes in individuals with PD. Statistical heterogeneity will be assessed through *I*^*2*^ statistics with a cut-off score of 50% using the ‘metafor’ package in R software.

#### Assessment of reporting biases

The methods section of the articles will be compared with the results section. If sufficient data is included, we will assess reporting bias by using a funnel plot.

#### Data synthesis

Data of the clinically important outcome measures from selected articles will be pooled to increase the overall sample size. This will produce a meta-analysis or descriptive review. If the studies are homogeneous and use the outcome measures reflecting activities of daily living performance, gait and walking, motor and cognitive function, or quality of life, variables will be statistically analysed. If outcome measures used in individual studies are heterogeneous and cannot be pooled for a meta-analysis, a descriptive review will analyse variance in interventions and outcomes.

If a meta-analysis is conducted, an analysis of the results on the outcome measures will be performed using post-intervention scores (means and SDs) to determine the overall effectiveness between the experimental and control interventions. An analysis of the long-term carryover effect post-intervention will also be conducted. The follow-up period is considered as the period following the initial post-intervention data collection. Corresponding authors will be contacted via email for original data where the published data was insufficient for data analysis.

All analysis will be performed using the ‘metafor’ package in R software, where the fixed effect or the random effect model with 95% CI is applied. Random effects models will be used, if the estimated effects in the included studies are not identical. A funnel plot can be included to detect bias in the meta-analysis results or systematic heterogeneity in the selected studies [29].

## Discussion

Given the impact of PD on individuals’ body structure and function, activities and participation, it is imperative to review the evidence regarding the effectiveness of imagery in the rehabilitation of people with PD. Therefore, the proposed systematic review will explore the efficacy of imagery as treatment in improving overall rehabilitation outcomes in individuals with PD. Imagery has a strong neuroscience base. It is a cost-effective and non-invasive treatment with limited adverse complications. As such, the outcomes of this review will guide clinical practice and inform future research in enhancing outcomes for people with PD. By conducting a systematic search and meta-analysis of available studies examining the effectiveness of imagery in populations with PD, this review will influence health care outcomes by providing best practice guidelines to promote and aid rehabilitation for people with PD.

This systematic review may be limited by a few factors. For example, most of the included studies may utilise imagery as part of the intervention. This may limit the examination of the effect of using imagery only on the reported outcomes. A report on the confidence in the cumulative evidence, such as using the Grading of Recommendations Assessment, Development, and Evaluation (GRADE) system, may be used. A structured approach for summarising the quality of evidence can be used in a clinical guideline.

## Data Availability

The datasets used and/or analysed during the current study are available from the corresponding author on reasonable request.

## Appendix

Table 1

**Table 1:** Sample database search strategy in Embase

Search line	Search
**1**	Parkinson disease/or parkinsonism/or hypokinesia/
**2**	(((Parkinson* or Neurodegenerative) adj2 disease*) or hypokinesia).ti,ab,kw.
**3**	Guided imagery/or imagery/or imagination/
**4**	(((motor or mental or visuomotor or visual motor or guided or imitation) adj4 imagery) or kinesthetic motor imagery or simulated movement* or neuro-cognitive).ti,ab,kw.
**5**	1 or 2
**6**	3 or 4
**7**	5 and 6

## Abbreviations

CI: Confidence interval
CINAHL: Cumulative Index to Nursing and Allied Health Literature
Embase: Embase Biomedical Answers
ICF: International Classification of Functioning
MEDLINE: Medical Literature Analysis and Retrieval System Online
MD: Mean difference
NNT: Number needed to treat
PD: Parkinson’s disease
PEDro: Physiotherapy Evidence Database
PRISMA-P: Preferred Reporting Items of Systematic Reviews and Meta-Analyses Protocol
RR: Risk ratio
